# The metabolic-immune interface of obesity in Covid-19: a role for angiotensin ii and inflammatory cytokines

**DOI:** 10.3389/fimmu.2025.1729494

**Published:** 2026-01-05

**Authors:** Cíntia Maria Rodrigues, Juliane Duarte Santos, Bruna Carolina Chaves Garcia, Marcelo Henrique Fernandes Ottoni, Karine Beatriz Costa, Marina Luiza Baêta Costa, Vívian Gonzalez Figueiredo, Daniel Macedo, Danilo Bretas de Oliveira, Etel Rocha Vieira, Evelin Capellari Cárnio

**Affiliations:** 1 Systemic Inflammation Physiology Laboratory, Postgraduate Degree in Fundamental Nursing, Ribeirão Preto School of Nursing, University of São Paulo, Ribeirão Preto, São Paulo, Brazil; 2 Nursing Department, Federal University of the Jequitinhonha and Mucuri Valleys, Diamantina, Minas Gerais, Brazil; 3 Postgraduate Program in Health Sciences, Faculty of Medicine, Federal University of the Jequitinhonha and Mucuri Valleys, Diamantina, Minas Gerais, Brazil; 4 Faculty of Medicine, University of the Jequitinhonha and Mucuri Valleys (UFVJM), Diamantina, Brazil

**Keywords:** angiotensin II, cytokines, interleukins, obesity, RAAS, SARS - COV - 2

## Abstract

**Background:**

Obesity is a major risk factor for severe COVID-19, partly explained by chronic systemic low-grade inflammation and renin-angiotensin-aldosterone system (RAAS) dysregulation.

**Objectives:**

To investigate the relationship between obesity and COVID-19 severity by measuring plasma angiotensin II (Ang II) and pro and anti-inflammatory cytokines across BMI categories.

**Methods:**

In a cross-sectional cohort of 142 adults (Lean and Obese), including mild and severe COVID-19 cases and matched uninfected controls. Plasma Ang II, IL-1β, IL-6, IL-10, and TNF were quantified by ELISA. Associations with BMI and clinical severity were assessed using ANOVA and correlation analyses.

**Results:**

Obese patients showed elevated Ang II, IL-1β, IL-6, and TNF, alongside reduced IL-10, compared to lean individuals and controls. Ang II positively correlated with BMI. Severe cases showed elevated neutrophil-to-lymphocyte ratios and greater need for ventilatory support requirements. Notably, mortality occurred exclusively among obese patients.

**Conclusion:**

Obesity exacerbates COVID-19 severity through RAAS imbalance and, amplified inflammatory responses. Ang II and pro-inflammatory cytokines may serve as early predictive biomarkers of disease progression in obese individuals, highlighting the metabolic-immune interface as a critical determinant of COVID-19 outcomes.

## Highlights

- Severe COVID-19 and death occur exclusively in obese patients.
- Obesity triggers RAAS imbalance with elevated Angiotensin II and hyperinflammation.
- Pro-inflammatory cytokines (IL-1β, IL-6, TNF) surge while IL-10 drops in obesity.
- Angiotensin II and inflammatory markers may predict COVID-19 severity early.

## 1 Introduction

The renin–angiotensin–aldosterone system (RAAS) is a central hormonal network regulating blood pressure and fluid homeostasis (1–3). The classical pathway of RAAS begins when the kidneys release the renin in response to low blood pressure or reduced sodium levels (4–6). Renin cleaves angiotensinogen into angiotensin I (Ang I), which is subsequently converted into angiotensin II (Ang II), primarily in the lungs by angiotensin-converting enzyme (ACE). Ang II binds to the angiotensin II type 1 receptor (AT1R), leading to vasoconstriction, stimulation of aldosterone release from the adrenal glands, promotion of renal sodium reabsorption, and increased fluid retention (1, 2, 4, 5, 7). These effects collectively raise blood pressure and circulating volume. While essential for physiological regulation, chronic overactivation of the classical RAAS pathway is strongly associated with cardiovascular complications (2).

Beyond this classical axis, the RAAS also comprises a protective counter-regulatory arm, in which Ang II or Ang I are metabolized by angiotensin-converting enzyme 2 (ACE2) to form angiotensin 1–7 (Ang 1–7). This heptapeptide exerts vasodilatory, anti-inflammatory, anti-fibrotic, and cardioprotective effects through activation of the Mas receptor (MasR) (8, 9). Ang 1–7 opposes many of the actions of Ang II/AT1R by promoting nitric oxide release, reducing oxidative stress, and inhibiting cell proliferation and cytokine production (8–10).

The balance between the two main arms of the renin–angiotensin–aldosterone system (RAAS)—the classical pathway (ACE/Ang II/AT1R) and the alternative pathway (ACE2/Ang 1–7/MasR)—is essential for maintaining cardiovascular and metabolic homeostasis (11–13). During SARS-CoV-2 infection, the viral spike (S) protein binds to the ACE2 receptor to enter host cells, particularly in the lungs, heart, kidneys, and gastrointestinal tract (14, 15). This interaction of the SARS-CoV-2 virus leads. ACE2 internalization and downregulation at the cell surface, resulting in reduced enzymatic activity and consequent accumulation of Ang II. Increased Ang II availability enhances AT1R-mediated responses, thereby promoting inflammation, oxidative stress, vasoconstriction, and thrombosis (14, 15).

Such dysregulation has been implicated in the pathogenesis and severity of COVID-19, especially among individuals with comorbidities such as hypertension, diabetes, or obesity (2, 5, 16). This pathological imbalance is further amplified by chronic non-communicable diseases (NCDs), with obesity exerting a particularly prominent effect (17). Obesity is recognized as a state of low-grade chronic inflammation – often referred to globally as “globesity” (14, 15) – and is strongly associated to more severe COVID-19 outcomes (18, 19). In individuals with obesity, prolonged viral persistence and an exacerbated inflammatory response, characterized by elevated levels of pro-inflammatory interleukins (e.g., IL-1β and IL-6), anti-inflammatory cytokines such as IL-10, tumor necrosis factor (TNF), and altered adipokine secretion, contribute to additional disruption of RAAS signaling, amplifying Ang II-mediated effects (5, 20).

Several studies have examined RAAS components in the plasma of COVID-19 patients, with a primary focus on Ang II levels in critically ill individuals. However, the findings remain inconsistent (5, 7, 10, 21–23), and no study has systematically compared RAAS activity across lean and obese populations. In this context, our results demonstrate that Ang II levels are elevated in individuals with obesity in direct association with disease severity. Furthermore, our data challenge a previously proposed hypothesis suggesting that severe illness and systemic dysregulation would hinder the detection of RAAS metabolites. In contrast, we were able to reliably quantify Ang II at picogram levels—even in non-infected individuals—at concentrations comparable to those reported by Reindl-Schwaighofer et al. (24).

## 2 Methods

### 2.1 Study design

**Figure 1:**
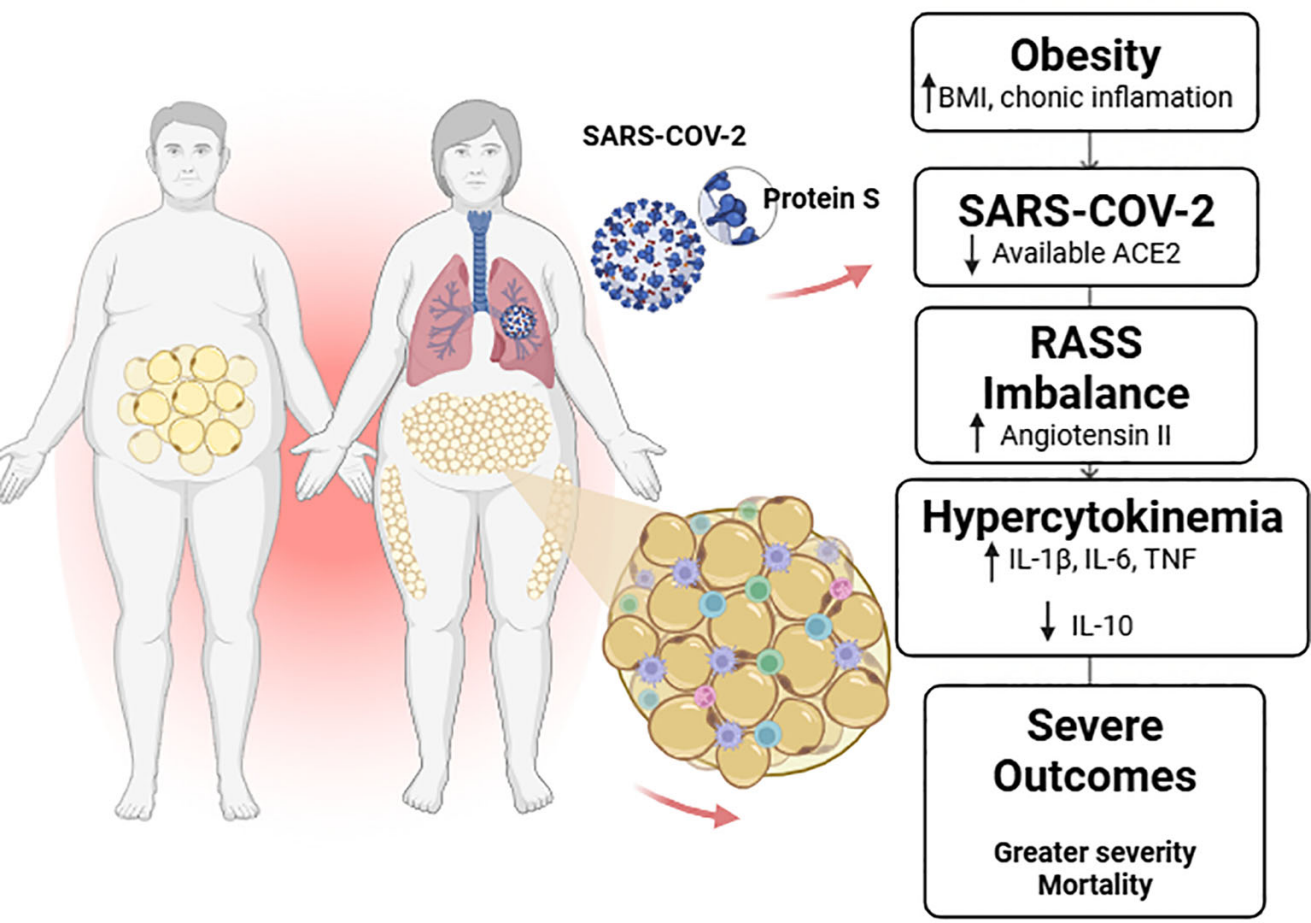
Representative diagram of how obesity and SARS-CoV-2 infection interact. Imbalance of the Renin–Angiotensin–Aldosterone System (RAAS), increased angiotensin II, release of pro-inflammatory cytokines, and reduced IL-10, resulting in worse clinical prognosis and mortality exclusively in obese patients. Image created with BioRender, 2025.

This was a cross-sectional cohort study involving adult patients of both sexes, aged 18 years or older, with a confirmed molecular diagnosis of SARS-CoV-2 infection by real-time RT-PCR. The study was approved by the Ethics Committees of the Federal University of the Jequitinhonha and Mucuri Valleys (UFVJM) and the Ribeirão Preto School of Nursing, University of São Paulo (EERP/USP), under the following protocols: 55519822.4.0000.5108, 34189420.2.0000.5108, 4.557.181 (for individuals with Influenza-like illness, i.e., mild symptoms), 4.202.959 (for patients with severe acute respiratory syndrome—SARS, moderate/severe conditions), and 4.533.696. **Figure 1** of this study was created using Biorender: Scientific Image and Illustration Software.

### 2.2 Setting

The study was conducted with partnership between UFVJM, EERP/USP, and the School Laboratory of Clinical Analysis (LEAC), encompassing 31 municipalities in the Jequitinhonha Valley macroregion. Clinical data were obtained from electronic medical records of the Santa Casa de Caridade de Diamantina and from primary care units (ViVver). Biological samples (nasopharyngeal swabs and peripheral blood) were collected between 2020 and 2024. A non-probabilistic, consecutive sampling strategy was employed to recruit patients with confirmed SARS-CoV-2 infection, either hospitalized or in home isolation, and a control group without infection. The sample size was determined based on the local population of Diamantina, Minas Gerais (47,825 inhabitants) (25).

### 2.3 Participants

Participants were categorized into two BMI-based groups—lean (LN) and obese (OB)—and further stratified by COVID-19 severity (mild *vs.* severe). The control group included BMI-matched individuals who tested negative for SARS-CoV-2.

The inclusion criteria comprised adults aged 18 years or older, classified by BMI, with SARS-CoV-2 infection time between 0 and 14 days, confirmed by RT-PCR testing. Negative controls, also confirmed by the same molecular test, were included. The exclusion criteria were: individuals who had recently received the vaccination (<30 days), patients with chronic kidney disease due to altered renin production, resulting in increased levels of angiotensin II and aldosterone, and patients with incomplete clinical data.

COVID-19 Diagnosis SARS-CoV-2 RNA was detected using qualitative real-time RT-PCR. Nucleic acid extraction was performed using Maxwell^®^ RSC 16 (Promega) and amplification using Applied Biosystems StepOne or StepOnePlus systems (ThermoFisher Scientific, USA). Target genes included N1 and N2 (2019-nCoV primer/probe sets). Samples with cycle threshold (Ct) value ≤40 were considered positive. Quantification of Cytokines and Adipokines

Plasma levels of IL-1β, IL-6, IL-10 and TNF were measured using commercial ELISA kits (DuoSet^®^, R&D Systems). All assays were conducted according to the manufacturer’s instructions, using human plasma samples stored at (2–8°C). Samples were analyzed in duplicate.

### 2.4 Angiotensin II measurement

Plasma Ang II concentrations were quantified using a specific ELISA kit (EIA Kit, SIGMA-ALDRICH, Merck; Catalog No. RAB0010). Blood samples were collected in pre-chilled tubes containing a protease inhibitor cocktail (Thermo Fisher 100X) to prevent peptide degradation. Plasma was processed within 3 hours and stored at –80°C. until analysis.

### 2.5 Statistical analysis

Data were analyzed using GraphPad Prism 8.0 and SPSS version 22.0. Quantitative variables were expressed as mean ± standard deviation or median (interquartile range), depending on distribution, which was assessed using the Kolmogorov-Smirnov test. Categorical variables were expressed as absolute and relative frequencies. Associations between BMI and variables such as symptoms, clinical aspects, comorbidities, and outcome were assessed using the chi-square test. Comparisons between groups were performed using one-way or two-way ANOVA followed by Tukey’s post-hoc test. Pearson’s correlation coefficients were calculated to assess the association between BMI and Ang II levels. Statistical significance was set at p ≤ 0.05.

## 3 Results

### 3.1 Demographic and clinical characterization by body composition using in individuals with COVID-19

This study evaluated 150 participants recruited from domiciliary settings, Emergency Care Units (UPAs), and Intensive Care Units (ICUs). Of these, 142 individuals were included in the final analysis; eight were excluded due to incomplete questionnaire responses or missing of clinical information in medical records.

**Table 1**, presents the epidemiological, clinical, and laboratory characteristics stratified by body composition according to BMI. The sample was sex-matched across control and COVID-19-positive groups, comprising 87 women (61.3%) and 55 men (38.7%). Among individuals with confirmed SARS-CoV-2 infection, 31 (21.8%) were classified as lean and 69 (48.6%) as obese—the latter representing the largest subgroup.

**Table 1:** Demographic and clinical characteristics by body mass index (BMI).

Variables	LN- (n = 22)	Control	LN + (n = 31)	COVID-19	Total (n = 142)	P value
		OB- (n = 20)		OB+ (n = 69)		
Sex ^(^*^n^*^%)^						
Woman	16 (18,3)	11(12,7)	18 (20,6)	42 (48,4)	87 (61,3)	0,0082
Men	6 (10,9)	9 (16,4)	13 (23,6)	27 (49,1)	55 (38,7)	
Age ^(mean ± SD)^	36,7 ± 9,9	40,8 ± 13,8	44,6 ± 20,8	44,5 ± 16,5	41,2 ± 15,2	0,0017*
Clinical aspects ^(mean ± SD)^						
Oxygen Saturation (SpO2) (%)	98,9 ± 0,8	98,3 ± 1,1	96,1 ± 2,5*	93,8 ± 3,5*/#	–	<0,0001***
Systolic Blood Pressure (SBP) (mmHg)	116,4 ± 13,8	120,8 ± 14	122,7 ± 14,7	126,2 ± 17,3	–	0,0244
Diastolic Blood Pressure (DBP) (mmHg)	71,7 ± 10,2	77,3 ± 9,7	75,7 ± 9,5	77,4 ± 10,7	–	0,0778
Heart Rate (BPM)	71,7 ± 12,3	77,4 ± 9,8	87,4 ± 12,5*	87,6 ± 12,7*	–	<0,0003*
Laboratory aspects ^(mean ± SD)^						
Hematocrit (%)	41,4 ± 4,9	42,9 ± 4,4	43,1 ± 7,5	41,9 ± 6	–	0,7768
Hemoglobin (g/dl)	14,9 ± 1,6	15,0 ± 1,7	14,7 ± 2,4	14,2 ± 2,3	–	0,1483
Leukocytes (K/µL)	5,9 ± 1,3	5,8 ± 1,8	6,1 ± 2,2	7,5 ± 3	–	0,0179*
Neutrophils (K/µL)	3,3 ± 1,4	3,4 ± 0,8	4,6 ± 3,3	4,8 ± 2,3	–	0,1569
Lymphocytes (K/µL)	2,2 ± 0,6	2,5 ± 0,5	2,6 ± 1,1	3,2 ± 2,7	–	0,2071
N/L Ratio (K/µL)	0,8 ± 0,4	0,8 ± 0,2	0,4 ± 0,4	1,9 ± 1,5*/#	–	<0,0001***

Source: Author (2025).

Categorical variables are expressed as frequencies (absolute - n, relative %). These are followed by cross-tabulation analysis using the Chi-square test, comparing BMI to comparing BMI to clinical and laboratory parameters. Continuous variables were calculated using the One-Way ANOVA test with Tukey’s *post hoc* test.

*Indicates significant differences in intergroup comparisons (positives versus negatives) to assess the effect of viral infection. LN- with LN+, OB- with OB+. # Denotes significant intragroup difference (positives only) to assess the effect of BMI, LN+ with OB+. The p-value represents an intergroup comparison of p < 0.05. Results below this value are considered highly significant and are expressed by the symbols * corresponding to p < 0.0003 and < 0.0005 and/or *** p < 0.0001. LN-, lean negative (control); OB-, obese negative (control). LN+, lean positive COVID-19; OB+, obese positive COVID-19.

Laboratory parameters supported the clinical observations. Individuals with obesity of both sexes exhibited reduced hemoglobin concentrations, a factor associated with dyspnea and tachycardia. They also showed decreased total leukocyte and lymphocyte counts, accompanied by elevated neutrophil-to-lymphocyte (NLR) ratio— an established biomarker of poor and increased mortality in COVID-19. These findings reinforce the hypothesis that obesity constitutes an independent risk factor for increased susceptibility to SARS-CoV-2 infection and is associated with a higher likelihood of severe clinical manifestations.

To explore the relationship between body composition and the prevalence of comorbidities, **Table 2** summarizes the most frequent conditions observed in the study cohort. Only individuals with mild or severe COVID-19 were included in this comparison due to the limited number of moderate cases. Systemic arterial hypertension emerged as the most prevalent comorbidity, followed by chronic obstructive pulmonary disease (COPD), dyslipidemia, and cardiovascular disease.

**Table 2:** Demographic and clinical characteristics, comorbidities, and outcomes according to disease severity and BMI.

Variables	LN (n=20)	Influenza-like Illness (ILI)	LN (n=12)	Severe acute respiratory syndrome (SARS)	Total (n=100)	P value
		OB (n=43)		OB (n=25)		
Sex ^(n %)^						
Female Male	15(24,6) 5 (12,8)	26 (42,6) 17 (43,6)	4 (6,6) 8(20,5)	16 (26,2) 9 (23,1)	61 39	<0,0001***
Age ^(mean ± SD)^	34,5 ± 11,2	39,3 ± 10,8	68,6 ± 14,4	62,2 ± 21,3	51,2 ± 14,4	<0,0001***
Signs and symptoms ^(n %)^						
Cough	6 (10,5)	25(43,8)	9 (15,8)	17 (29,8)	57	0,344
Runny nose	13 (31)	20 (47,6)	2 (4,8)	7 (66,6)	42	0,593
Fever	5 (14,3)	4 (31,4)	7 (20)	12(34,2)	35	0,452
Headache	5 (12,8)	15 (66,6)	3 (7,7)	5 (12,8)	39	0,548
Anosmia	14 (27,5)	25 (49,0)	3 (5,8)	9 (17,6)	51	0,350
Dysgeusia	8 (21,1)	23 (60,5)	2 (5,2)	5 (13,2)	38	0,571
Dyspnea	3 (7,7)	11 (28,2)	9 (23,1)	6 (41,0)	39	0,449
Desaturation	16 (13,0)	1 (2,2)	10(21,8)	19 (41,3)	46	0,667
Comorbitidies ^(n %)^						
Hypertension	3 (12,0)	6 (24,0)	7 (28,0)	9 (36,0)	25	0,587
Dyslipidemia	2 (13,3)	8 (53,3)	0 (0)	5 (33,4)	15	0,005*
Diabetes *Mellitus*	0 (0)	3 (50,0)	2 (33,4)	1 (16,6)	6	0,196
Respiratory disease	1 (9,1)	2 (18,2)	2 (18,2)	6 (54,5)	11	0,449
Cancer	0 (0)	0 (0)	1 (33,3	2 (66,6)	3	0,504
Mental Illness	1 (11,0)	4 (44,5)	0 (0)	4 (44,5)	9	0,169
Clinical outcome ^(^*^n^*^%)^						
Recovery	20 (22,2)	43 (47,8)	9 (10,0)	18 (20,0)	90	0,256
Death	0 (0)	0 (0)	2 (20,0)	8 (80,0)	10	

Source: Author (2025).

Categorical variables are expressed as frequencies (absolute - n, relative %). These are followed by cross-tabulation analysis using the Chi-square test, comparing BMI to signs and symptoms, comorbidities, and clinical outcome.

*Indicates significant differences in intergroup comparisons (positives versus negatives) to assess the effect of viral infection. LN- with LN+, OB- with OB+. # Denotes significant intragroup difference (positives only) to assess the effect of BMI, LN+ with OB+. The p-value represents an intergroup comparison of p < 0.05. Results below this value are considered highly significant and are expressed by the symbols * corresponding to p < 0.0003 and < 0.0005 and/or *** p < 0.0001. LN-, lean negative (control); OB-, obese negative (control). LN+, lean positive COVID-19; OB+, obese positive COVID-19.

### 3.2 Factors associated with severity in COVID-19 patients

Focusing on disease severity, **Table 3** depicts a cyclical clinical profile in which obesity may contribute to the development of comorbidities, while SARS-CoV-2 infection in obese individuals further alters physiological parameters, aggravating their health status. Systolic and diastolic blood pressure were slightly elevated in hospitalized patients with severe disease, including those without COVID-19, indicating that obesity alone was sufficient to alter these parameters. Heart rate showed an upward trend across all infected groups. Interestingly, ventilatory variables showed that oxygen saturation in critically ill patients dropped significantly, consistent with the findings reported above. Patients in this group also required non-invasive ventilation and, in many cases, invasive mechanical ventilation through intubation. These observations highlight the impaired lung expansion and compromised ventilatory capacity in critically ill individuals—particularly patients with obesity, who exhibited poorer outcomes.

**Table 3:** Baseline laboratory and blood gas variables according to COVID-19 severity.

Variables	ILI (DP)-*^n^* (%) (n = 59)	SARS (DP)-*^n^* (%) (n = 48)	P value
BMI (kg/m2)	25,9 ± 5,3	31,7 ± 5,8	<0,0001***
Systolic Blood Pressura (mmHg)	126,38 ± 19,71	128, 33 ± 9,83	<0,0001***
Diastolic Blood Pressura (mmHg)	72,85 ± 12,51	78, 33 ± 9, 83	<0,0001***
Heart Rate (bpm)	–	84,93 ± 14,99	–
Oxygen Saturation (%)	94,84 ± 1,55	90,90 ± 6,0	0,1838
Ventilatory parameters			
Mechanical Ventilation n (%) Round-glass Opacity on Imaging (Chest CT)	2 (3,4) 7 (11,9)	12 (25) 33 (68,8)	
Labatory tests ^(mean ± SD)^			
Hematocrit (%)	31,59 ± 21,80	22,87 ± 20,65	0,0417
Hemoglobin (g/dL)	10,64 ± 7,75	7,64 ± 6,88	0,0475
Leukocytes (10³/µl)	4,58 ± 3,77	5,41 ± 4,09	0,2643
Neutrophils(10³/µl)	3,74 ± 2,75	4,46 ± 3,51	0,2354
Lymphocytes (10³/µl)	2,71 ± 2,38	7,88 ± 8,36	<0,0001***
Neutrophil/Lymphocyte Ratio (NLR)	0,29 ± 1,18	0,32 ± 0,58	0,8481
Platelets (10³/µl)	129 ± 125	85,96 ± 145,5	0,0850
CRP (mg/L)	0	81,15 ± 31,86	–
D-Dimer (ng/mL)	0	44,22 ± 166,4	–
Arterial blood gas ^(mean ± SD)^			
Blood pH	–	7,4 ± 2,8	–
Oxygen Pressure (pO2)	–	75,5 ± 28,4	–
Carbon Dioxide Pressure (pCO2)	–	43,8 ± 14,4	–
Bicarbonate (HCO_3_)	–	26,3 ± 6,0	–
Sodium (Na - mEq/L)	–	140 ± 6,9	–
Potassium (K - mEq/L)	–	1,2 ± 1,0	–
Creatinine (mg/dL)	–	1,2 ± 1,0	–
Urea (mg/dL)	–	45,4 ± 24,1	–
Lactate Dehydrogenase (LDH) (U/L)	–	573,1 ± 309,8	–
Clinical outcome ^(n%)^			
Recovery Death	65 (100) 0	35 (73) 10 (20,8)	<0,0001***

Source: Author (2025).

Categorical variables are expressed as frequencies (absolute - n, relative %). Cross-tabulation analysis is followed by the Chi-square test, comparing BMI to clinical and laboratory parameters in ILI and SARS patients.

*Indicates significant differences in intergroup comparisons (positives versus negatives) to assess the effect of viral infection. LN- with LN+, OB- with OB+. # Denotes significant intragroup difference (positives only) to assess the effect of BMI, LN+ with OB+. The p-value represents an intergroup comparison of p < 0.05. Results below this value are considered highly significant and are expressed by the symbols * corresponding to p < 0.0003 and < 0.0005 and/or *** p < 0.0001. LN-, lean negative (control); OB-, obese negative (control). LN+, lean positive COVID-19; OB+, obese positive COVID-19.

As shown in **Table 3**, patients with severe COVID-19 (requiring hospitalization) presented laboratory abnormalities such as reduced hematocrit and hemoglobin, thrombocytopenia, leukocytosis, lymphocytosis, and neutrophilia. The increase in the neutrophil-to-lymphocyte ratio (NLR), an inflammatory biomarker, with values above 2.21, is a predictor of ICU hospitalization and is associated with an 8% higher risk of death compared to those with mild disease (managed at home), being positively correlated with worse prognosis. Biochemical parameters such as C-reactive protein, D-dimer and arterial blood gas values, although altered, were assessed only in critically ill patients, in accordance with SUS hospital care priority protocols, and therefore it was not possible to compare. Regarding clinical outcome all patients with mild disease achieved complete recovery, whereas among severe cases, 7 patients (16.7%) died, all of whom belonged to the obese BMI group. This finding demonstrates a positive association between obesity and worse outcomes in COVID-19.

### 3.3 Overactive cytokine immune response

**Figure 2:**
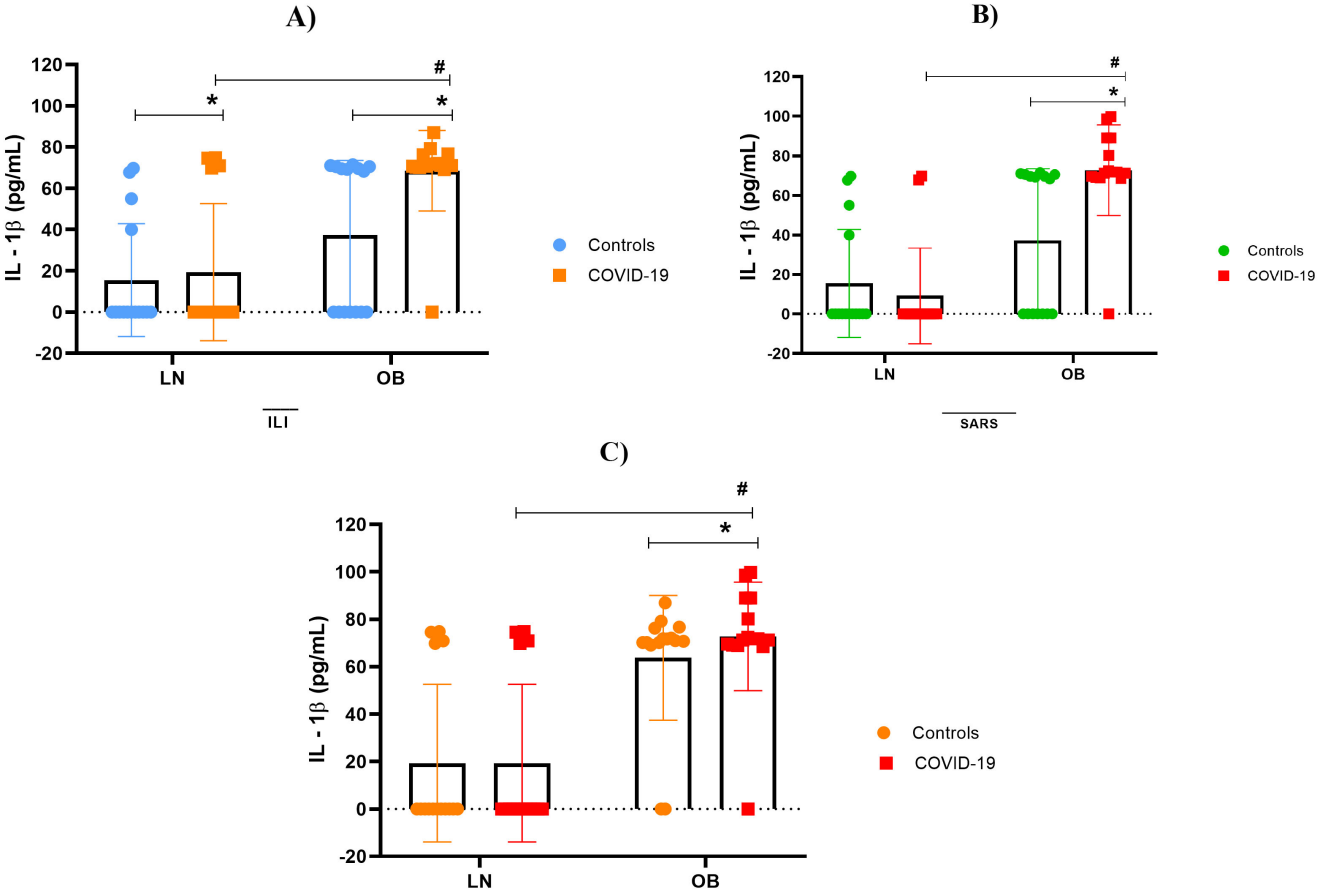
Interleukin 1β concentration. **(A)** IL-1β stratified by BMI, between controls and mild cases with flu-like illness; **(B)** IL-1β stratified by BMI, between controls and severe cases with severe acute respiratory syndrome; **(C)** IL-1β comparison between flu-like illness and severe acute respiratory syndrome. Source: Author (2025). **(A–C)** were calculated using the Two-Way ANOVA test with Tukey’s post-hoc test. * Indicates significant differences in intergroup comparisons (positives versus negatives) to assess the effect of viral infection. LN- with LN+, OB- with OB+. # Denotes significant intragroup difference (positives only) to assess the effect of BMI, LN+ with OB+ p < 0.05. LN-, negative lean (control); OB-, negative obese (control). LN+, lean positive COVID-19; OB+, obese positive COVID-19. SG, Flu-Like Syndrome (mild/moderate manifestations); SARS, Severe Acute Respiratory Syndrome (severe manifestations).

**Figure 3:**
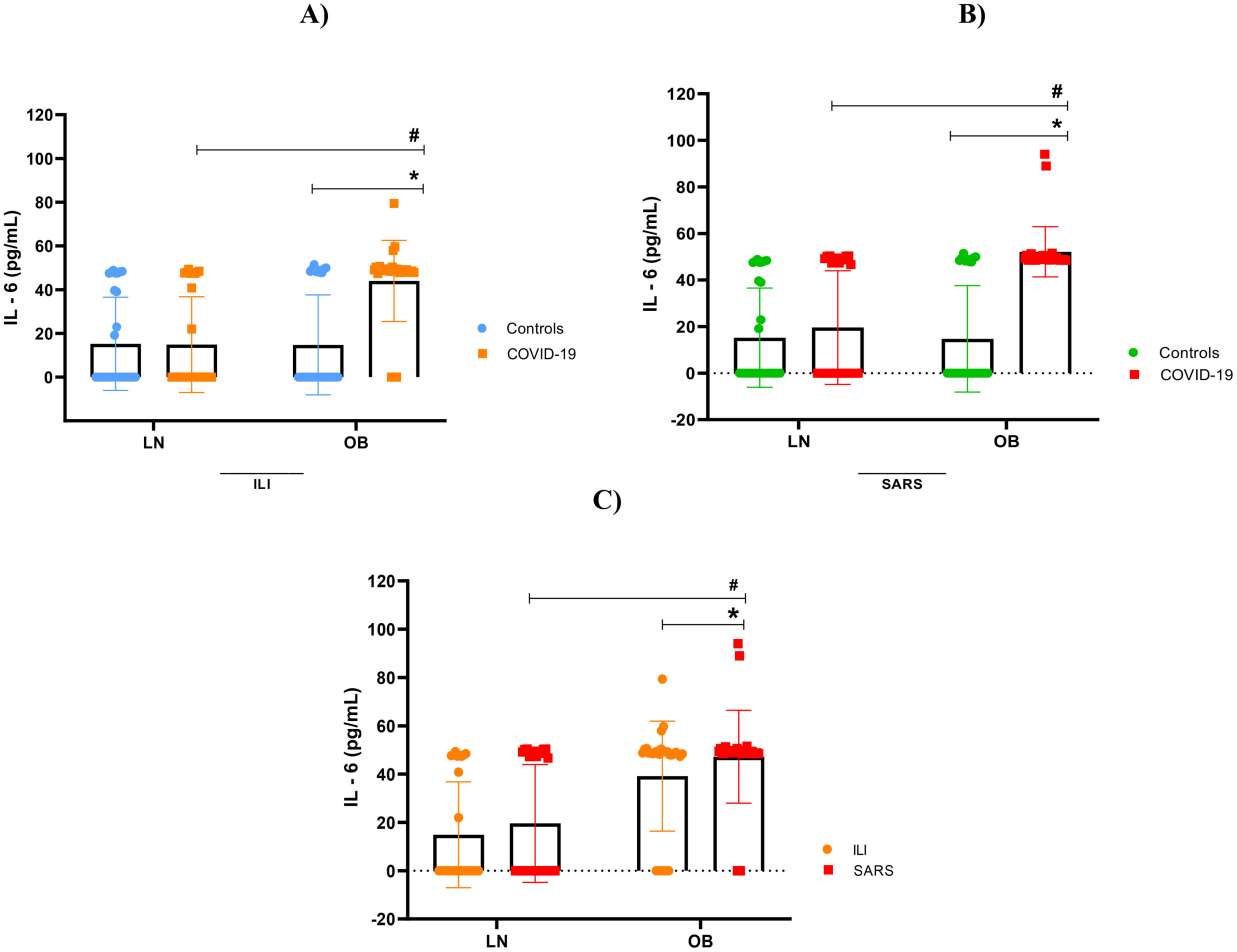
Interleukin-6 concentration. **(A)** IL-6 stratified by BMI, between controls and mild cases with flu-like illness; **(B)** IL-6 stratified by BMI, between controls and severe cases with severe acute respiratory syndrome; **(C)** IL-6 comparison between flu-like illness and severe acute respiratory syndrome. Source: Author (2025). **(A–C)** were calculated using the Two-Way ANOVA test with Tukey’s post-hoc test. * Indicates significant differences in intergroup comparisons (positives versus negatives) to assess the effect of viral infection. LN- with LN+, OB- with OB+. # Denotes significant intragroup difference (positives only) to assess the effect of BMI, LN+ with OB+ p < 0.05. LN-, negative lean (control); OB-, negative obese (control). LN+, lean positive COVID-19; OB+, obese positive COVID-19. SG, Flu-Like Syndrome (mild/moderate manifestations); SARS, Severe Acute Respiratory Syndrome (severe manifestations).

**Figure 4:**
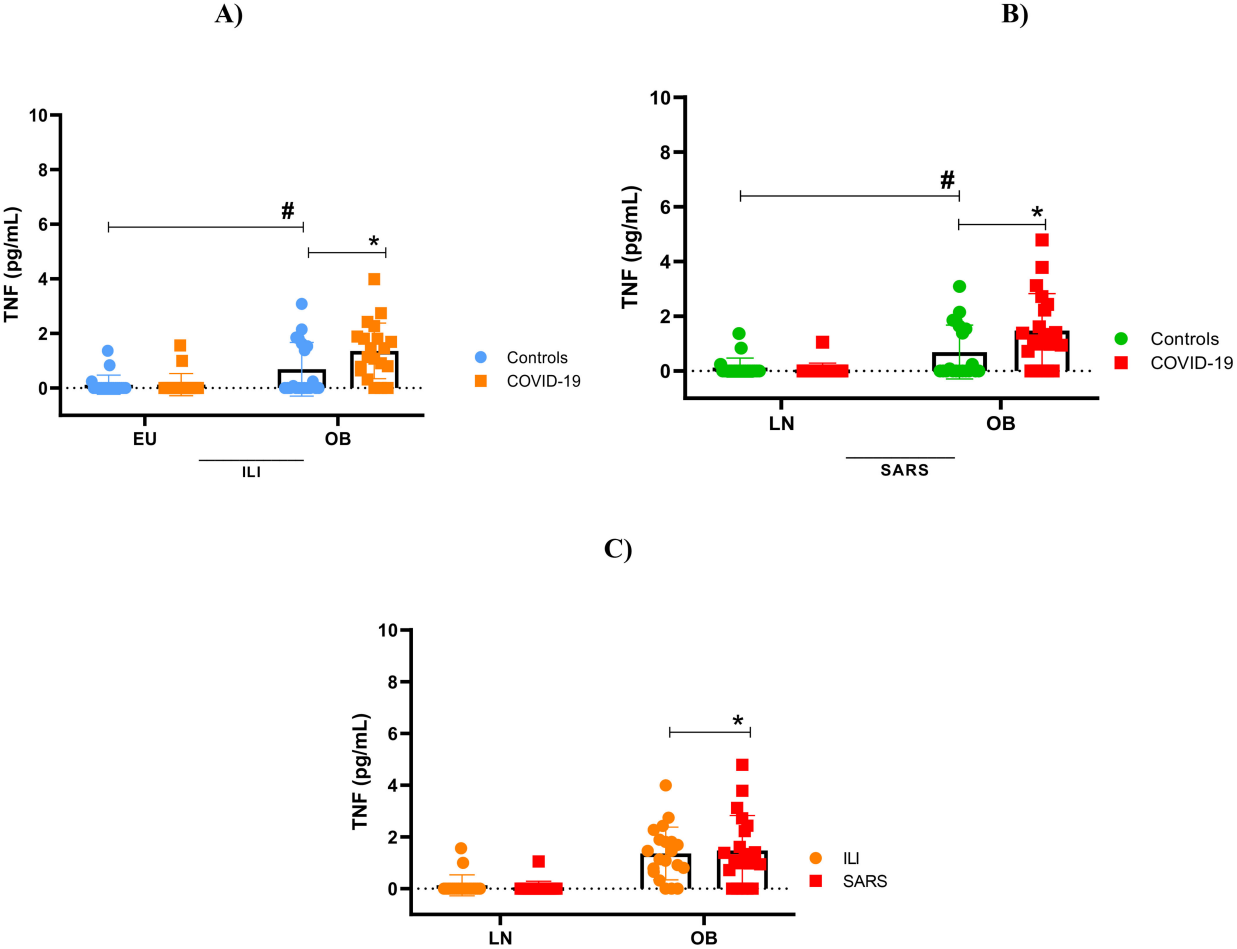
Tumor necrosis factor (TNF) concentration. **(A)** TNF stratified by BMI, between controls and mild cases with flu-like illness; **(B)** TNF stratified by BMI, between controls and severe cases with severe acute respiratory syndrome; **(C)** TNF comparison between flu-like illness and severe acute respiratory syndrome. Source: Author (2025). **(A–C)** were calculated using the Two-Way ANOVA test with Tukey’s post-hoc test. * Indicates significant differences in intergroup comparisons (positives versus negatives) to assess the effect of viral infection. LN- with LN+, OB- with OB+. # Denotes significant intragroup difference (positives only) to assess the effect of BMI, LN+ with OB+ p < 0.05. LN-, negative lean (control); OB-, negative obese (control). LN+, lean positive COVID-19; OB+, obese positive COVID-19. SG, Flu-Like Syndrome (mild/moderate manifestations); SARS, Severe Acute Respiratory Syndrome (severe manifestations).

**Figure 5:**
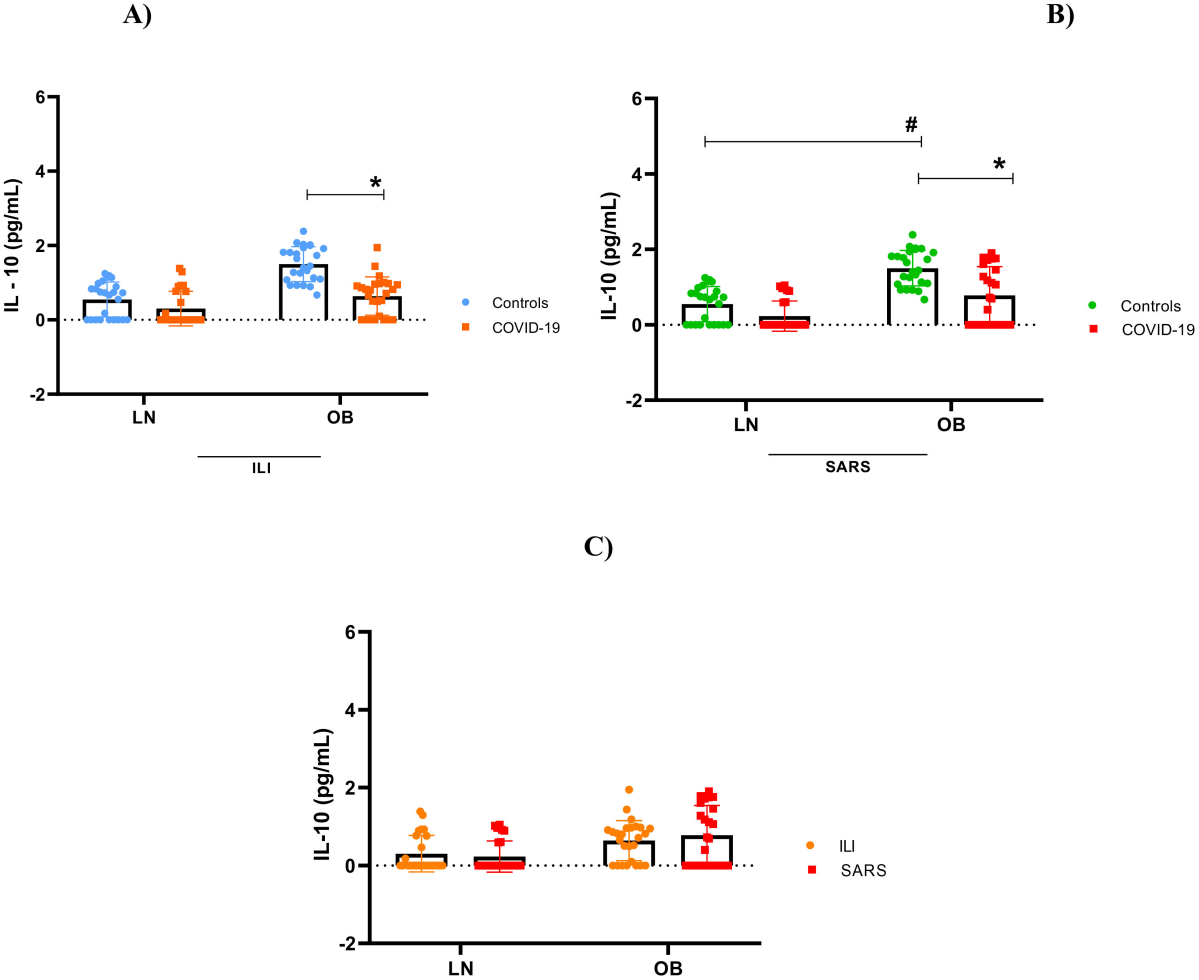
Interleukin - 10 concentration. **(A)** IL-10 stratified by BMI, between controls and mild cases with flu-like illness; **(B)** IL-10 stratified by BMI, between controls and severe cases with severe acute respiratory syndrome; **(C)** IL-10 comparison between flu-like illness and severe acute respiratory syndrome. Source: Author (2025). **(A–C)** were calculated using the Two-Way ANOVA test with Tukey’s post-hoc test. * Indicates significant differences in intergroup comparisons (positives versus negatives) to assess the effect of viral infection. LN- with LN+, OB- with OB+. # Denotes significant intragroup difference (positives only) to assess the effect of BMI, LN+ with OB+ p < 0.05. LN-, negative lean (control); OB-, negative obese (control). LN+, lean positive COVID-19; OB+, obese positive COVID-19. SG, Flu-Like Syndrome (mild/moderate manifestations); SARS, Severe Acute Respiratory Syndrome (severe manifestations).

Our findings indicate immunological impairment in this population, characterized by cytokine hyperactivity, hypercytokinemia, observed both in obese patients with mild clinical symptoms and, more prominently, in those with severe obesity. As shown in **Figures 2**–**5**, levels of IL-1β, IL-6, TNF and IL-10 were significantly higher levels in obese individuals, compared to lean subjects, with even greater elevations in patients worse clinical outcomes.

### 3.4 A positive correlation between plasma Ang II levels and obesity is associated with greater clinical severity in individuals infected with COVID-19

**Figure 6:**
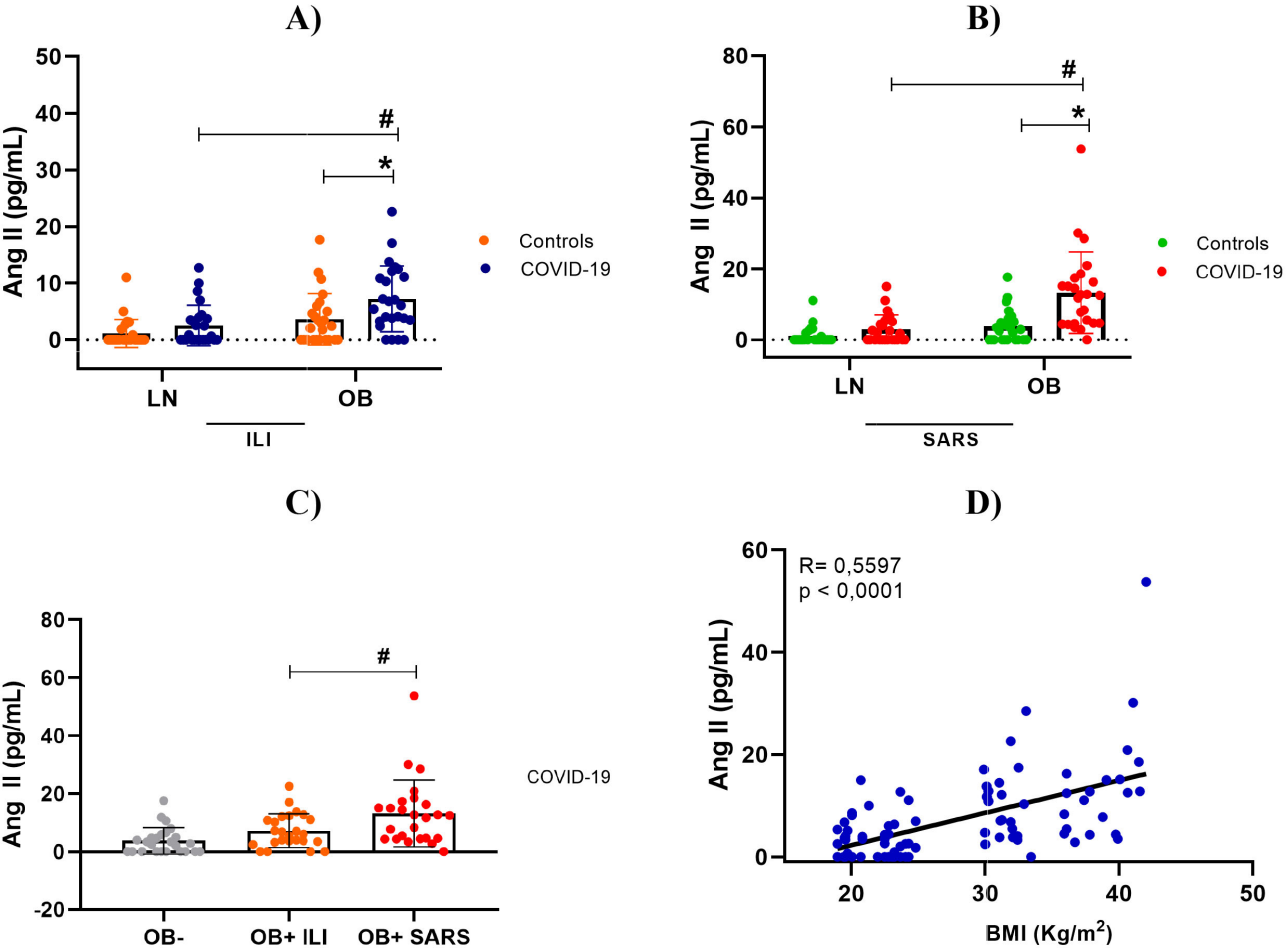
Plasma angiotensin II levels. **(A)** Lean and stratified by BMI, between controls and mild cases with flu-like illness; **(B)** Lean and obese individuals with SARS, stratified by BMI, between controls and severe cases with severe acute respiratory syndrome; **(C)** Comparison of Angiotensin II levels between obese individuals (control and COVID-19), in both severity levels. **(D)** Correlation between BMI and Angiotensin II concentration. Source: Author (2025). **(A–C)** were calculated using two-way ANOVA with Tukey’s post-hoc test. **(D)** was calculated using Pearson’s correlation. * Indicates significant differences in intergroup comparisons (positive versus negative) to assess the effect of viral infection. LN- with LN+, OB- with OB+. # Denotes significant intragroup difference (positives only) to assess the effect of BMI, LN+ with OB+ p < 0.05. LN-, lean negative (control); OB-, obese negative (control). LN+, lean positive for COVID-19; OB+, obese positive for COVID-19. SG, Influenza-like syndrome (mild/moderate manifestations); SARS, Severe acute respiratory syndrome (severe manifestations).

Plasma Ang II levels (pg/mL) were measured in all COVID-19 patients and control subjects. The analysis included a clinical subdivision, in which lean and non-severe obese patients were compared with their controls, and patients with severe conditions with their respective/matched controls. Interestingly and consistent with the findings of Liu et al. (21) and Wu et al. (26) plasma levels of Ang II in obese patients with COVID-19 were significantly higher than those in non-severe negative and reaching even higher values in individuals with severe disease (**Figure 6**).

To determine whether the elevation of angiotensin II could be associated with obesity, we performed a comparison between mild and severe obese COVID-19–positive patients, and Pearson’s correlation between Ang II and BMI. As expected, there was a positive correlation between plasma angiotensin II levels and higher BMI, indicating that obesity may alter the RAAS axis, leading to hyperinflammation and greater disease severity.

## 4 Discussion

Our study demonstrates that obesity substantially exacerbates COVID-19 severity by disrupting the RAAS and amplifying inflammatory responses (27, 28). Obese individuals infected with SARS-CoV-2 exhibited elevated plasma levels of Ang II, IL-1β, IL-6, and TNF, alongside reduced IL-10 concentrations, indicating a hyperinflammatory and immunologically dysregulated state (17, 29, 30). Moreover, the positive association between Ang II levels BMI supports the hypothesis that excess adiposity enhances RAAS activation and contributes to disease progression (11, 31, 32).

Obesity, also known as Adiposity-Based Chronic Disease (ABCD), is increasingly recognized as a chronic immunometabolic disease characterized by persistent endocrine, metabolic, and inflammatory disorders (4, 33). During the COVID-19 pandemic, obesity emerged as one of the strongest predictors of severity, with individuals presenting a BMI ≥30 kg/m² (4, 11, 34) demonstrating a markedly greater need for intensive care and mechanical ventilation (19, 35, 36). Thus, obesity should be considered not only a comorbidity but also a biological condition that amplifies the host’s vulnerability to viral pathogens.

The coexistence of chronic systemic inflammation and SARS-CoV-2 infection establishes a milieu that predisposes individuals to immune dysregulation and RAAS perturbation (4). Adipose tissue expresses angiotensinogen and other RAAS components, and excess adiposity enhances Ang II production through adipocyte hypertrophy, increased free fatty acids, and high-fat dietary patterns (37–39). Moreover, the upregulation of ADAM17, frequently observed in obesity, promotes ACE2 shedding and TNF release, further aggravating inflammation (40, 41). Collectively, these alterations precondition individuals with obesity to stronger RAAS activation, oxidative stress, and endothelial dysfunction even prior to viral exposure (5, 7, 10, 24, 26, 42–44).

Upon SARS-CoV-2 infection, these vulnerabilities become more pronounced. Viral engagement of ACE2 reduces its availability on the cell surface, shifting RAAS signaling (45, 46) toward the classical ACE/Ang II/AT1R axis (8, 47–49) and weakening the counterregulatory Ang-(1–7)/MasR and alamandine/MrgD pathways (50, 51). As a result, Ang II accumulates and drives vasoconstriction, mitochondrial dysfunction, NF-κB activation, and widespread endothelial injury (7, 11, 52). Concomitantly, the reduction in ACE2-derived peptides diminishes anti-inflammatory control, facilitating uncontrolled cytokine production (28, 31, 53). TLR-mediated recognition of viral components further fuels innate immune activation (38, 54), triggering synthesis of IL-1β, IL-6, TNF, and IFN-γ (55, 56), and establishing a feed-forward inflammatory loop strongly implicated in severe COVID-19 (30, 57, 58).

Within this immunometabolic landscape, the synergistic interplay between obesity and SARS-CoV-2 becomes evident. In our cohort, individuals with obesity showed elevated plasma Ang II, heightened concentrations of IL-1β, IL-6, and TNF, and reduced IL-10, supporting the presence of a dysregulated inflammatory state exacerbated by viral infection (48, 59, 60). The positive association between Ang II and BMI reinforces the mechanistic link between adiposity and RAAS hyperactivation (5, 61, 62). Furthermore, although women predominated in our sample—reflecting global patterns of higher obesity prevalence—no significant sex differences in Ang II levels were observed. This may be explained by the near-menopausal age of many participants, a period in which protective effects of estradiol and progesterone on ACE2 and AT2R signaling begin to diminish (63, 64). Thus, hormonal status, age, and adiposity appear to collectively shape RAAS responsiveness during SARS-CoV-2 infection (24, 43).

These immunometabolic interactions are further compounded by cardiometabolic comorbidities commonly associated with obesity (65). Hypertension, metabolic syndrome, and dyslipidemia converge mechanistically on oxidative stress, endothelial dysfunction, and RAAS activation (7, 66). Hypertriglyceridemia and elevated LDL promote lipid deposition and vascular remodeling, accelerating atherogenesis and contributing to endothelial vulnerability during viral infection (65, 67). In addition, lipid raft enrichment of ACE2 facilitates SARS-CoV-2 entry, suggesting that dyslipidemia may not only represent a comorbidity but also potentiate viral infectivity (2, 67–69). Consequently, the constellation of cardiometabolic alterations in individuals with obesity strengthens the biological rationale linking adiposity to more severe COVID-19 outcomes (67, 70–72).

Clinically, the inflammatory markers observed in our cohort provide further support for this mechanistic model. Leukocytosis, neutrophilia, elevated neutrophil-to-lymphocyte ratio (NLR), and increased D-dimer levels correlated with severity, aligning with prior studies identifying these markers as prognostic indicators (21, 40, 73–75). The progression of cytokine elevation and coagulopathy between days 7 and 10 of symptom onset—previously associated with mortality—was consistent with the systemic inflammatory signaling induced by heightened Ang II activity (21, 23, 48, 49, 59, 76). Although lymphocyte count and CRP showed inconsistent associations across studies, our findings underscore the value of immunometabolic biomarkers, particularly Ang II, NLR, and D-dimer, in risk stratification (32, 48, 77–79).

Finally, although Ang-(1–7) (80) and alamandine were not measured, our preliminary data point to a trend of reduced expression of MasR and MrgD receptors (50), which implies that obesity and SARS-CoV-2 infection may contribute to decreased protective RAAS signaling (4, 40, 81). Taken together, our findings provide compelling evidence for a dual-hit model in which obesity establishes a primed inflammatory and RAAS-altered baseline, and SARS-CoV-2 amplifies these disruptions, culminating in severe immunometabolic imbalance (5, 7, 43). This framework advances current understanding of COVID-19 pathophysiology and reinforces the need to consider adiposity-driven biological mechanisms when evaluating risk and therapeutic strategies.

The limitations of this study include the presence of cardiometabolic comorbidities in all body composition groups, which may have acted as confounding factors and reduced our ability to isolate the specific contribution of obesity. Furthermore, the absence of important metabolic markers—such as leptin, adiponectin, resistin, and insulin—and the lack of direct measurements of Ang-(1-7), aldosterone, and renin limited a more detailed assessment of the immunometabolic pathways and the RAAS involved. Future perspectives of the study will be to conduct cardiovascular and metabolic biomarkers to further deepen the mechanistic understanding of obesity-related vulnerability to SARS-CoV-2.

## 5 Conclusion

This study identified obesity as a key biological factor that exacerbated COVID-19, demonstrating a direct association between increased BMI, elevated Ang II, and heightened pro-inflammatory cytokines, alongside reduced IL-10. By quantifying Ang II across different BMI categories and clinical severity levels, including mild and non-hospitalized cases, we provide mechanistic insights into the metabolic-immune interface of obesity. Adiposity-related, inflammatory, and RAAS markers emerge as promising targets for clinical assessment and potential therapeutic interventions.

## 6 Novelty

This study reveals that obesity markedly worsens COVID-19 severity through dysregulation of the renin–angiotensin–aldosterone system (RAAS) and a hyperinflammatory response. We observed a strong positive correlation between body mass index (BMI) and plasma Ang II levels, indicating that excess visceral adiposity amplifies RAAS activation and disease progression. Obese patients also showed elevated IL-1β, IL-6, TNF, reduced IL-10, higher neutrophil-to-lymphocyte ratios (NLR), and an increased need for ventilatory support, with adverse outcomes—including mortality—occurring predominantly in this population. These findings provide a mechanistic explanation, supporting a dual-hit model in which obesity primes immunometabolic vulnerability that is exacerbated by SARS-CoV-2 infection.

## 7 Significance

This study provides novel mechanistic evidence that obesity exacerbates COVID-19 severity through RAAS dysregulation and heightened inflammatory responses, with a direct correlation between BMI and Ang II levels. Elevated plasma Ang II, IL-1β, IL-6, and TNF, alongside reduced IL-10, identify potential biomarkers for early risk stratification. Clinically, obese patients exhibited higher NLR, greater ventilatory requirements, and mortality restricted to this group, underscoring their vulnerability. These findings advance the understanding of adiposity as an active endocrine contributor to infectious disease outcomes and highlight translational opportunities for risk assessment and therapeutic strategies targeting RAAS and inflammation.

## Data Availability

The datasets presented in this study can be found in online repositories. The names of the repository/repositories and accession number(s) can be found in the article/supplementary material.
